# Nitric Oxide Functions as a Signal in Ultraviolet-B-Induced Baicalin Accumulation in *Scutellaria baicalensis* Suspension Cultures

**DOI:** 10.3390/ijms15034733

**Published:** 2014-03-18

**Authors:** Jin-Jie Zhang, Xue-Qin Li, Jun-Wei Sun, Song-Heng Jin

**Affiliations:** 1 School of Marine Science, Ningbo University, Ningbo 315211, Zhejiang, China; E-Mail: zhangjinjie@nbu.edu.cn; 2 Tianmu College, Zhejiang A&F University, Zhuji 311800, Zhejiang, China; E-Mail: lxqin@zafu.edu.cn; 3 Department of Biology, College of Life Sciences, China Jiliang University, No. 258 Xueyuan Road, Hangzhou 310018, Zhejiang, China

**Keywords:** baicalin, nitric oxide, NOS activity, *Scutellaria baicalensis*, UV-B irradiation

## Abstract

Stress induced by ultraviolet-B (UV-B) irradiation stimulates the accumulation of various secondary metabolites in plants. Nitric oxide (NO) serves as an important secondary messenger in UV-B stress-induced signal transduction pathways. NO can be synthesized in plants by either enzymatic catalysis or an inorganic nitrogen pathway. The effects of UV-B irradiation on the production of baicalin and the associated molecular pathways in plant cells are poorly understood. In this study, nitric oxide synthase (NOS) activity, NO release and the generation of baicalin were investigated in cell suspension cultures of *Scutellaria baicalensis* exposed to UV-B irradiation. UV-B irradiation significantly increased NOS activity, NO release and baicalin biosynthesis in *S. baicalensis* cells. Additionally, exogenous NO supplied by the NO donor, sodium nitroprusside (SNP), led to a similar increase in the baicalin content as the UV-B treatment. The NOS inhibitor, *N*^ω^-nitro-l-arginine (LNNA), and NO scavenger, 2-(4-carboxyphenyl)-4,4,5,5-tetramethylimidazoline-1-oxyl-3-oxide (cPTIO) partially inhibited UV-B-induced NO release and baicalin accumulation. These results suggest that NO is generated by NOS or NOS-like enzymes and plays an important role in baicalin biosynthesis as part of the defense response of *S. baicalensis* cells to UV-B irradiation.

## 1. Introduction

Ultraviolet-B (UV-B) irradiation can negatively affect plant growth, photosynthesis and pigmentation [1,2]. Plants have developed a variety of defense strategies in response to UV-B irradiation. For example, plant cells synthesize a number of secondary metabolites, including flavonoids, to mitigate UV-B damage [3–5]. Therefore, UV-B irradiation can be used as an effective method to increase the yield of useful secondary metabolites in plant cell cultures.

Nitrogen monoxide or nitric oxide (NO) is a free radical gas formed endogenously in many biological systems, including animals, plants and microbes. NO has a wide range of biological activities. Although the physiological functions of NO in plants remain to be fully characterized, evidence is emerging that NO plays a regulatory role in plant growth, development, defense responses and seed dormancy [6–10]. As the production of NO in plant tissues and cells usually occurs in response to pathogen invasion [11], fungal elicitors [12] and abiotic stresses [13–15], it is possible that NO plays a prominent role in the signaling and regulation of plant defense or stress responses. In animal systems, most NO is synthesized by nitric oxide synthase (NOS) [16], and there is increasing evidence to support the existence of NOS activity in plant systems [17].

**Figure 1.**
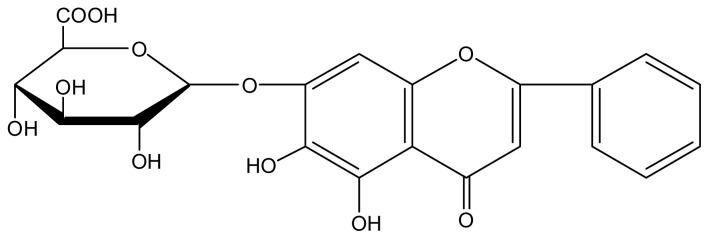
The chemical structure of baicalin (5,6-dihydroxy-flavone-7-*O*-glucuronide).

*Scutellaria baicalensis*, also known as Chinese skullcap, has been used to treat allergic and inflammatory diseases in China since ancient times. *S. baicalensis* is also known to contain numerous flavone derivatives, including baicalin, baicalein and wogonin [18]. Among them, baicalin (Figure 1) has been widely used for the treatment of various diseases, such as hepatitis, pneumonia, allergies, diabetes and cancer [19,20]. Previous studies have demonstrated that baicalin possesses a wide range of biological and pharmacological activities, such as anti-inflammatory and anti-cancer properties [21,22], and provides potent free radical scavenging and xanthine oxidase inhibition [23], thus improving endothelial function and conferring cardiovascular protective effects [24,25].

NOS-synthesized NO has been proven to be involved in both the upregulation of the chalcone synthase gene and inhibition of pea stem elongation in response to UV-B irradiation [26,27]. Involvement of NO in UV-B-induced activation of flavonoid synthesis has been reported in *Ginkgo biloba* [28] and *Betula pendula* [29]. Therefore, NO may play a key role in UV-B-induced plant secondary metabolite synthesis [5,30]. However, the molecular basis of the UV-B signaling cascades leading to the stimulation of flavonoid (especially baicalin) synthesis in plant cells has remained basically unknown until now. In the present study, *Scutellaria baicalensis* cells were used to investigate the effects of NO on the biosynthesis of baicalin, a plant polyphenolic compound. The main objective of this work was to identify the possible function of NO in the response of *S. baicalensis* cells to UV-B irradiation.

## 2. Results and Discussion

### 2.1. Results

**Figure 2.**
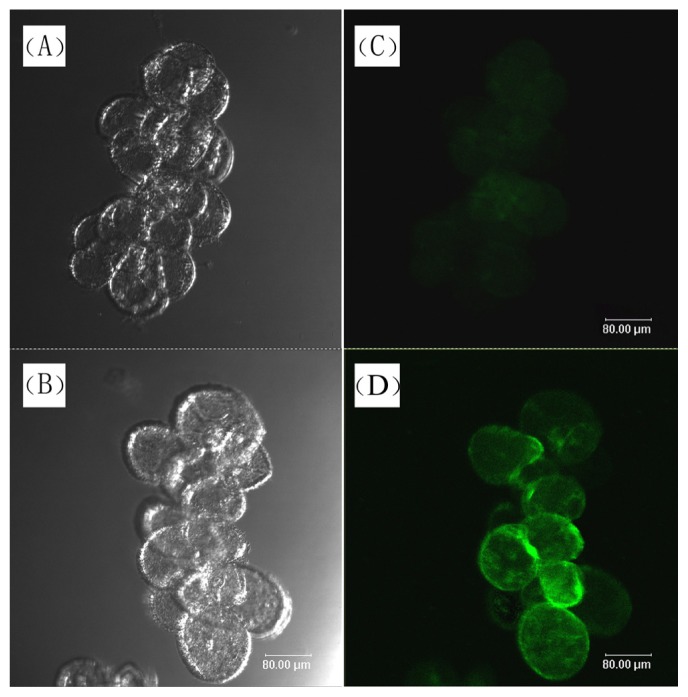
Visualization of NO generation in *S. baicalensis* cells. (**A**) Bright-field image of control cells without 4-amino-5-methylamino-2′,7′-difluorofluorescein (DAF-FM) staining; (**B**) Bright-field image of UV-B irradiated cells without DAF-FM staining; (**C**) Fluorescence microscopy (488 nm excitation and 510 nm emission) of DAF-FM-stained control cells without UV-B treatment; (**D**) Fluorescence microscopy of DAF-FM-stained cells after three days of 20 kJ m^−2^ d^−1^ UV-B irradiation. All cells were from seven-day-old agitated flask cultures.

**Figure 3.**
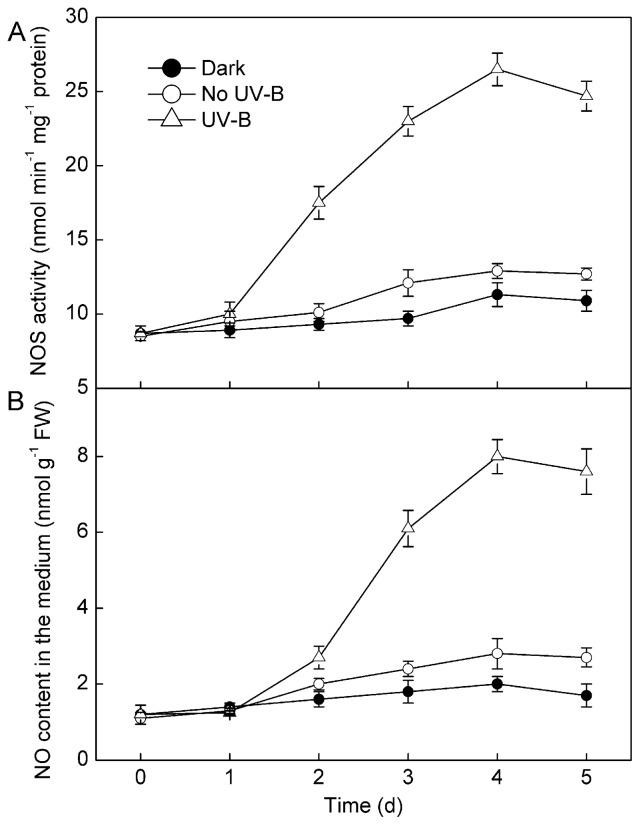
The time course of nitric oxide synthase (NOS) activity (**A**) and NO production (**B**) in *S. baicalensis* cells irradiated with 20 kJ m^−2^ d^−1^ UV-B or no UV-B for five days. Control cells were cultured in the dark. Data are the means ± SE of three replicates. FW, fresh weight.

The induction of intracellular NO production by UV-B irradiation in *S. baicalensis* cells could be directly observed by detecting the green fluorescence of 4-amino-5-methylamino-2′,7′-difluorofluorescein (DAF-FM) diacetate-stained cells via fluorescence microscopy. NO production was effectively stimulated by UV-B irradiation (Figure 2). The NOS activity and NO content of UV-B-treated cells were also significantly higher than that of cells without UV-B irradiation and dark-cultured cells, with the maximal NOS activity and NO content reached after a four-day treatment (Figure 3). Subsequently, NOS activity decreased, and the NO content did not increase further.

**Figure 4.**
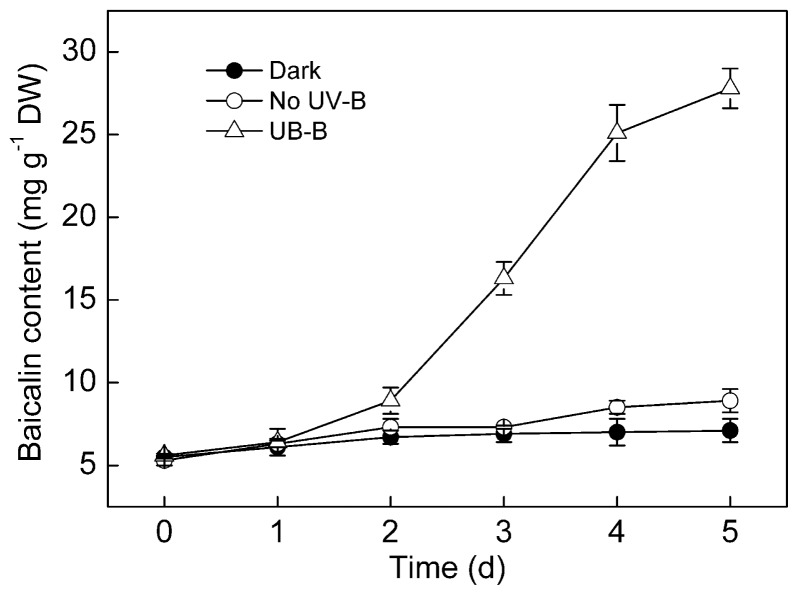
The time course of baicalin production by *S. baicalensis* cells irradiated with 20 kJ m^−2^ d^−1^ UV-B or no UV-B for five days. Control cells were cultured in the dark. Data are the means ± SE of three replicates. DW, dry weight.

UV-B irradiation stimulated baicalin production in *S. baicalensis* cell cultures, leading to a rapid increase in the baicalin content (Figure 4). The baicalin content of the UV-B-treated cell cultures reached a maximal level after five days of treatment, at which time the baicalin content was 3.1-fold higher than that of cells without UV-B irradiation. NOS activity and NO release reached maximal levels after four days of UV-B irradiation (Figure 3), while baicalin accumulation did not peak until Day 5 of treatment.

**Figure 5.**
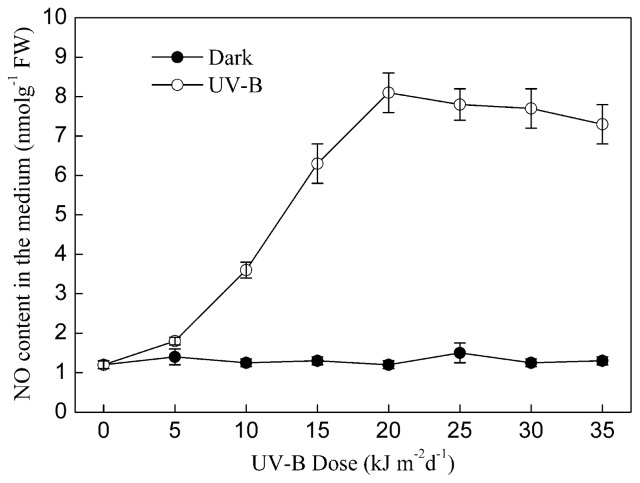
The effect of the dose of UV-B on NO accumulation in *S. baicalensis* cells. Seven-day-old cultures of *S. baicalensis* cells were treated with different doses of UV-B irradiation (UV-B). Control cells were cultured in the dark. The amount of NO was measured after four days of UV-B irradiation. Data are the means ± SE of three replicates.

**Figure 6.**
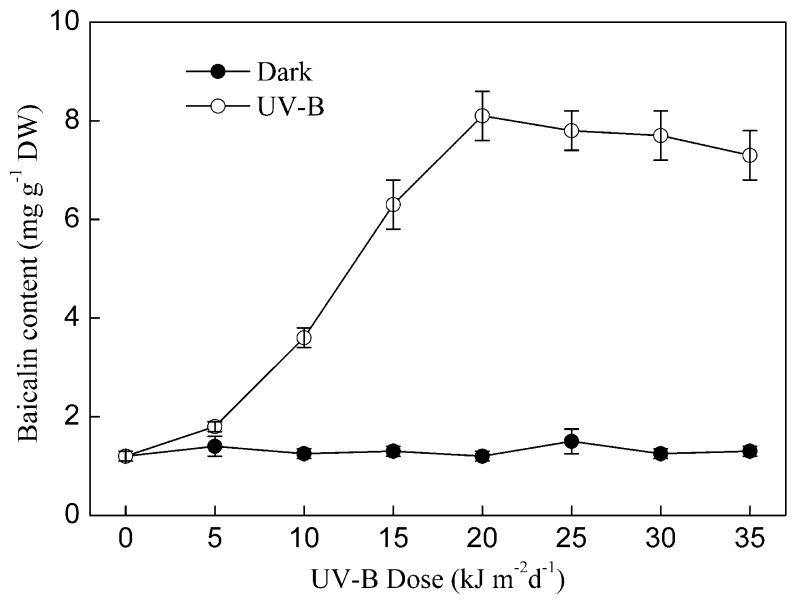
The effect of the dose of UV-B on baicalin production by *S. baicalensis* cells. Seven-day-old *S. baicalensis* cells were treated with different doses of UV-B irradiation (UV-B). The control cells were cultured in the dark. Baicalin content was determined after five days of UV-B irradiation. Data are the means ± SE of three replicates.

UV-B-induced NO and baicalin generation in *S. baicalensis* cells was dose-dependent. As shown in Figures 5 and 6, the content of NO and baicalin increased linearly with the dose of UV-B irradiation within the range 0–20 kJ m^−2^ d^−1^. However, both the NO content and baicalin content did not significantly increase further when the dose of UV-B irradiation exceeded 20 kJ m^−2^ d^−1^.

**Figure 7.**
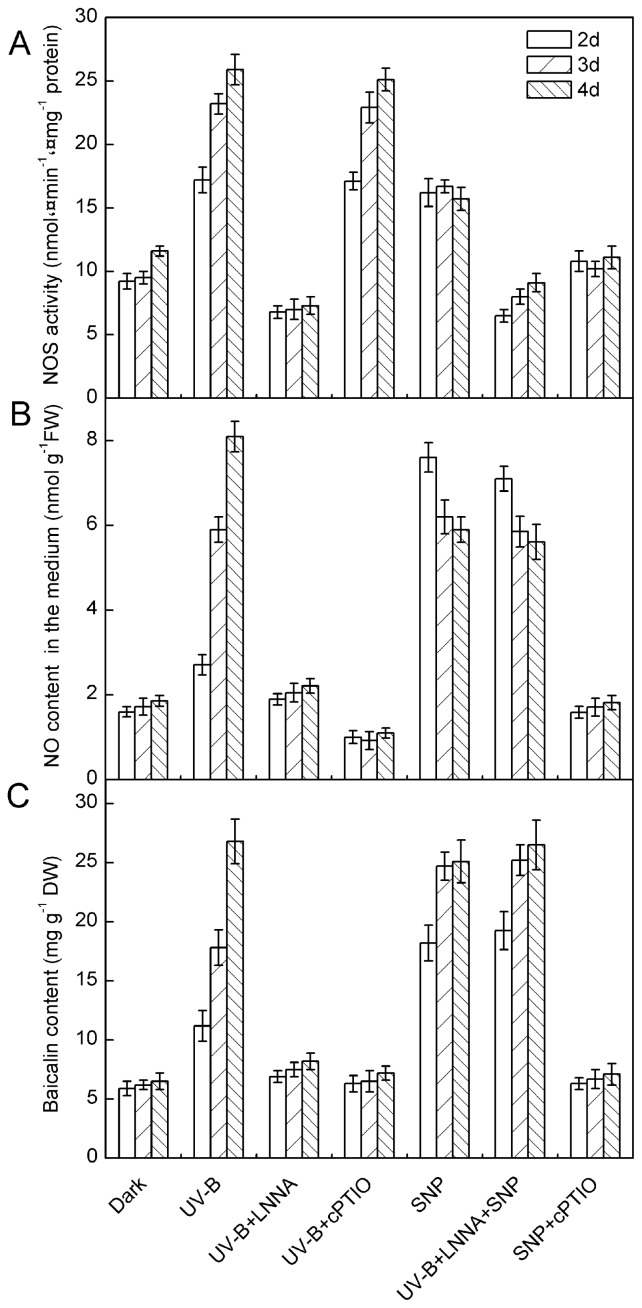
NOS activity (**A**); NO release (**B**) and baicalin content (**C**) in *S. baicalensis* cells irradiated with 20 kJ m^−2^ d^−1^ UV-B in the presence and absence of a NO scavenger, an NO donor and/or an NOS inhibitor. Five-day-old cultures were treated with 100 μmol L^−1^ of *N*^ω^-nitro-l-arginine (LNNA) (NOS inhibitor) with UV-B irradiation (UV-B + LNNA), 200 μM L^−1^ of cPTIO (NO scavenger) with UV-B irradiation (UV-B + cPTIO), 0.5 mM L^−1^ of sodium nitroprusside (SNP) (NO donor; SNP), 100 μM L^−1^ of LNNA and 0.5 mM L^−1^ of SNP with UV-B irradiation (UV-B + LNNA + SNP) or 0.5 mM L^−1^ of SNP and 200 μM L^−1^ of cPTIO (SNP + cPTIO). LNNA and cPTIO were added 30 min before UV-B treatment, and SNP was added at the same time as UV-B irradiation. The control cells were cultured in the dark and received the same volume of vehicle solvents only. Data are the means ± SE of three replicates.

Compared to control cultures, *N*^ω^-nitro-L-arginine (LNNA) (a NOS inhibitor) and c2-(4-carboxyphenyl)-4,4,5,5-tetramethylimidazoline-1-oxyl-3-oxide (cPTIO) (a NO-specific scavenger) significantly inhibited UV-B-induced NO release and baicalin synthesis in *S. baicalensis* cells (Figure 7), and UV-B-induced NOS activity was remarkably attenuated by LNNA, but not by cPTIO. These results strongly suggest that the NOS enzyme capable of NO generation exists in *S. baicalensis* cells and that NO is required for baicalin production in response to UV-B irradiation.

Sodium nitroprusside (SNP) was used as an NO donor in this work to investigate the effects of exogenous NO on baicalin production in *S. baicalensis* cells. In the absence of UV-B irradiation, the addition of 0.5 mmol L^−1^ SNP stimulated baicalin production in *S. baicalensis* cells, with this effect equivalent to up to 80% of the UV-B-induced response. These results suggest that NO alone is sufficient to trigger baicalin synthesis in *S. baicalensis* cells. The finding that the inhibitory effect of LNNA on UV-B-induced baicalin synthesis could be mitigated by the addition of SNP (0.5 mmol L^−1^) also supports this conclusion (Figure 7).

### 2.2. Discussion

The present study demonstrates that UV-B irradiation increases NOS activity and induces NO and baicalin generation in *S. baicalensis* cells. Direct application of NO using SNP could also induce baicalin synthesis (Figure 7). Additionally, UV-B-induced baicalin synthesis could be blocked by a NO scavenger or NOS inhibitor (Figure 7). These results suggest that NO is essential for UV-B-induced baicalin synthesis in *S. baicalensis* cells. UV-B induced upregulation of NO release accompanied with the UV-B-induced activation of baicalin synthesis (Figures 3 and 4), which indicates that NO may function upstream of baicalin synthesis in the response to UV-B irradiation.

It is widely believed that the synthesis of a variety of secondary metabolites contributes greatly to the defense responses of plants to biotic or abiotic stresses [31]. Certain receptors in plant cells recognize and interact with UV light [32–34], and UV-B-induced defense responses in plants usually activate the general phenylpropanoid and flavonoid glycoside pathways [3]. Flavonoids are regarded as the major metabolic response of cultured parsley cells to UV-B irradiation [35,36] and are also believed to be UV-B absorbing compounds [37]. Flavonoids are able to absorb UV radiation, reducing the risk of ROS generation [38,39]. It has been documented that UV-B irradiation induces both flavonoid accumulation and NO generation [29,37]. As a flavonoid with two pro hydroxyl groups, baicalin showed strong antioxidant activities [40]. In this study, the effect of NO on baicalin synthesis in *S. baicalensis* cells may be associated with the ability of NO to activate the defense responses of plant cells, as numerous studies have demonstrated that NO is an important signaling molecule involved in mediating the defense response of plants to UV-B irradiation [5–7]. Therefore, the baicalin biosynthetic pathway may be activated by NO as a result of NO triggering the stress responses of *S. baicalensis* cells. Additionally, the dose and saturation effects of UV-B-induced NO and baicalin generation in *S. baicalensis* cells observed in this work suggest that the defense responses of *S. baicalensis* cells to UV-B can reach a receptor saturation limit: once the appropriate receptor(s) were saturated by high levels of UV-B, additional doses of UV-B did not induce higher levels of NO and baicalin generation.

To date, the source of NO in plants remains poorly understood. In animals, NO biosynthesis is primarily catalyzed by the enzyme, NOS, which oxidizes *L-*Arg to *L-*citrulline and NO. In plants, NO can be synthesized by either enzymatic catalysis or an inorganic nitrogen pathway. During the last few years, several groups have provided evidence for the existence of NOS-like activity in plants. NOS-like activities have been identified in several plant species, and inhibitors of mammalian NOS have been reported to suppress elicitor-induced NO generation in *Arabidopsis*, soybean, tobacco, pea and *Taxus chinensis* [41–44]. An *Arabidopsis* NOS gene (*AtNOS1*) that regulates growth and hormonal signaling has been identified, and the protein activity of AtNOS1 can be inhibited by an inhibitor of mammalian NOS [45]. In addition, immunological analysis suggests that NOS-like proteins probably localize in the cytoplasm of maize [46], the peroxisomes of pea and olive [47] and the hypocotyls of sunflower [48]. Corpas *et al*. [49] found that endogenous NO was mainly localized in the vascular tissues of pea plants using a confocal laser scanning microscopy technique with the fluorescent probe, 4,5-diaminofluorescein diacetate, and confirmed the subcellular localization of NO in peroxisomes by electron paramagnetic resonance spectroscopy with the spin trap Fe(MGD)_2_ and fluorometric analysis with 4,5-diaminofluorescein diacetate. Recently, a novel NOS was identified in the green alga, *Ostreococcus tauri*. Its protein sequence shares 45% similarity to human NOS, and its catalytic domains share a high similarity to animal NOS in structural models. Interestingly, purified recombinant *O. tauri* NOS also possesses NOS activity *in vitro* [50]. These studies suggest that NOS enzymes may exist in plants. In the present study, UV-B-induced NO generation in *S. baicalensis* cells was strongly inhibited by the NOS inhibitor, LNNA, which suggests that NOS or NOS-like enzymes exist in *S. baicalensis* cells and can catalyze UV-B-induced NO generation.

In summary, the results of this work suggest that NO, generated by NOS or NOS-like enzymes, is involved as an important signal molecule in mediating UV-B-induced baicalin synthesis in *S. baicalensis* cells. The positive effect of UV-B irradiation on the synthesis of the active constituent in traditional Chinese medicine plants is intriguing. Therefore, the application of UV-B irradiation may offer a promising approach for producing baicalin by enhancing NO generation.

## 3. Experimental Section

### 3.1. Plant Cell Culture

The *S. baicalensis* plants used in this study were grown in Zhejiang Province, People’s Republic of China. A *S. baicalensis* cell line was induced from young stems incubated on MS medium [51] supplemented with 0.186 mg L^−1^ α-naphthaleneacetic acid (NAA), 2.253 mg L^−1^ 6-benzylaminopurine (BA), 30 g L^−1^ sucrose and 7 g L^−1^ agar. The callus culture line had been in culture for 5 months by the time this study was performed. Suspension cultures of the cell line were initiated from the callus culture in liquid media similar to that used for the callus culture excluding agar. Prior to use, the media was adjusted to pH 5.8 and sterilized by autoclaving at 121 °C for 20 min. Suspension cultures (100 mL) were maintained in 250 mL sterile Erlenmeyer flasks capped with Magenta B-Caps (Sigma-Aldrich, St. Louis, MO, USA) and incubated on an orbital shaker incubator at 25 °C with shaking at 100 rpm in the dark room. The suspension cultures were subcultured every 12 days. The callus and cell line used in this study maintained stable growth rates and morphological characteristics.

### 3.2. Experimental Conditions

Enhanced UV-B irradiation was generated with a filtered West Lake (Hangzhou Lamp Factory, Hangzhou, China) 30-W fluorescent sunlamps (290–320) following a published procedure [52]. The lamps were suspended above and perpendicular to the flasks, and the light was filtered using either 0.13 mm-thick pre-solarized cellulose diacetate filters (transmission down to 290 nm) for UV-B irradiation or 0.13 mm polyester film (transmission down to 320 nm) for no UV-B treatments. The desired intensity of irradiation was obtained by altering the distance between the lamps and the top of the flasks. The spectral irradiance from the lamps was determined using an Optronic Model 742 spectroradiometer (Optronic Labs., Orlando, FL, USA), weighted with the generalized plant response action spectrum [53] and normalized at 300 nm to obtain the desired level of biologically-effective UV-B irradiation.

Sodium nitroprusside (SNP; BDH, Poole, UK) was used as an NO donor. *N*^ω^-nitro-l-arginine (LNNA; Sigma-Aldrich, St. Louis, MO, USA) was used as an NO inhibitor and 2-(4-carboxyphenyl)- 4,4,5,5-tetramethylimidazoline-1-oxyl-3-oxide (cPTIO; Sigma-Aldrich, St. Louis, MO, USA) as an NO scavenger. These NO reagents and their concentrations were chosen on the basis of previous studies [54]. Both LNNA (100 μmol L^−1^) and cPTIO (200 μmol L^−1^) were added to the cultures 30 min prior to UV-B irradiation, and SNP (0.5 mmol L^−1^) was added at the start of UV-B irradiation.

### 3.3. Measurement of NO Production

NO accumulation in the culture media and cell extracts (see below) was assayed by monitoring the conversion of oxyhemoglobin to methemoglobin [55]. The assay was performed on 1-mL aliquots of the culture media, which were incubated for 5 min with 100 U catalase and 100 U superoxide dismutase to remove reactive oxygen intermediates before the addition of oxyhemoglobin. The assay was performed on 100 μL of the cell extracts, corresponding to 0.1 g fresh weight (FW). After 10 min of incubation with oxyhemoglobin (10 μM final concentration), the changes in the absorbance of the culture media and cell extracts were measured at 421 and 401 nm using a Shimadzu UV-1700 spectrophotometer (Shimadzu Corporation, Kyoto, Japan), and the NO levels were calculated using an extinction coefficient of 77 mM^−1^ cm^−1^ (A_401_ (methemoglobin) − A_421_ (oxyhemoglobin)).

NO was visualized using the NO-reactive cell-permeable fluorescent probe, 4-amino-5- methylamino-2′,7′-difluorofluorescein (DAF-FM) diacetate (Alexis Biochemicals, Lausanne, Switzerland), which fluoresces not only at neutral to basic pH, but also at acidic pH [56]. The cells were collected by gentle centrifugation (1 min at 500× *g*), resuspended in 10 μmol L^−1^ DAF-FM diacetate, incubated for 15 min at room temperature and then observed using a Leica DMIRE2 confocal laser scanning microscope equipped with Leica TCS-NT software and fluorescein isothiocyanate filter sets (Leica microsystems, Wetzlar, Germany). The data acquisition settings (laser power, pinhole size, scan conditions, detector settings, *etc*.) were identical for all experiments.

### 3.4. NOS Activity Assay

Crude enzyme extracts were prepared for the NOS activity assay according to Ninnemann and Maier [57] with modifications. The treated cells were homogenized in 10 mL of homogenization buffer (50 mM triethanolamine hydrochloride, pH 7.5, containing 0.5 mM ethylene-diamine tetraacetic acid, 1 μM leupeptin, 1 μM pepstatin, 7 mmol L^−1^ glutathione and 0.2 mmol L^−1^ phenylmethylsulfonyl fluoride). After centrifugation at 10,000× *g* for 20 min at 4 °C, the NOS activity of the supernatants was analyzed using the hemoglobin assay, as previously described [55].

### 3.5. Baicalin Determination

The high-performance liquid chromatography (HPLC) method described in our previous study [58] was used to determine the baicalin content of the cellular material. Briefly, 0.1 g dry cell powder was extracted twice with methanol, and the extracted solutions were combined and concentrated under vacuum. The residues were dissolved in 10 mL of methanol and analyzed by HPLC using a Shimadzu LC-6A apparatus with a Shimadzu C-R3A integrator and SPD-6A UV detector monitoring at 280 nm; Kromasil C18 column (dimensions, 250 mm × 4.6 mm; packing, 5 μm; Alltech, Deerfield, IL, USA). Samples were eluted with a mobile phase of methanol-0.5% phosphoric acid (50:50) at a flow rate of 1 mL min^−1^. Quantification of baicalin was based on an external baicalin standard (Sigma).

### 3.6. Statistical Analysis

All data is presented as the mean of at least three replicates. Statistical analysis was performed using Duncan’s multiple range test with Statgraphic^®^ Plus Professional Version 5 (2000) software (Manugistics Inc., Rockville, MD, USA).

## 4. Conclusions

In the present study, we used *Scutellaria baicalensis* cells to investigate the effects of nitric oxide (NO) on the biosynthesis of baicalin, a plant secondary metabolite. The main objective of this work is to identify the possible function of NO in the response of *S. baicalensis* cells to ultraviolet-B (UV-B) irradiation. Our findings suggest that NO is generated by nitric oxide synthase (NOS) or NOS-like enzymes and plays an important role in baicalin biosynthesis as part of the defense response of *S. baicalensis* cells to UV-B irradiation.
